# Differences between Young and Older Adults in the Control of Weight Shifting within the Surface of Support

**DOI:** 10.1371/journal.pone.0098494

**Published:** 2014-06-03

**Authors:** Elisabeth A. de Vries, Simone R. Caljouw, Milou J. M. Coppens, Klaas Postema, Gijsbertus J. Verkerke, Claudine J. C. Lamoth

**Affiliations:** 1 Center for Human Movement Sciences, University of Groningen, University Medical Center Groningen, Groningen, The Netherlands; 2 Department of Rehabilitation Medicine, Center for Rehabilitation, University of Groningen, University Medical Center Groningen, Groningen, The Netherlands; 3 Dept of Biomechanical Engineering, University of Twente, Enschede, The Netherlands; Ludwig-Maximilian University, Germany

## Abstract

An important reason for falling in elderly is incorrect weight-shifting. In many daily life activities quick and accurate weight-shifting is needed to maintain balance and to prevent from falling. The present study aims to gain more insight in age-related differences in the control of weight-shifting. Nine healthy older adults (70.3±6.9 years) and twelve young adults (20.9±0.5 years) participated in the study. They performed a weight shifting task by moving the body's center of pressure, represented by a red dot on a screen, in different directions, towards targets of different sizes and at different distances projected on a screen. Movement time, fluency and accuracy of the movement were determined. Accuracy was quantified by the number of times the cursor hit the goal target before a target switch was realized (counts on goal) and by the time required to realize a target switch after the goal target was hit by the cursor for the first time (dwelling time). Fluency was expressed by the maximal deviation of the performed path with respect to the ideal path and the number of peaks, or inflections in the performed path. Significant main effects of target size, target distance and age on all outcome measures were found. With decreasing target size, increasing target distance and increasing age, movement time significantly increased and fluency and accuracy significantly decreased (i.e. increased number of peaks, maximal deviation, number of times on the goal target and longer dwelling time around the goal target). In addition, significant interaction effects of size*age and distance*age were found. Older adults needed more time to perform the weight-shifting task and their movements were less fluent and accurate compared to younger adults, especially with increasing task difficulty. This indicates that elderly might have difficulties with executing an adequate adaptation to a perturbation in daily life.

## Introduction

Falls are a serious health problem among older adults, because the incidents are high and they lead to severe consequences. Each year one third of the people aged 65 years or older falls at least once [1]. In the upcoming decades, worldwide the aging population will increase, which will result in an increase in the amount of injuries due to a fall in older adults. Many daily life activities pose a risk to fall for older adults. For example, when reaching for a cup on a table, standing up from a chair or climbing the stairs, balance is disturbed by changes in posture, related changes in joint torques and environmental factors [2]. To maintain balance during these activities, people need to adequately adjust their posture, otherwise a fall is likely to occur. Due to the deterioration in neuromuscular, musculoskeletal and sensory systems that come with age, older adults have more difficulties in controlling their posture [3]. The deterioration in postural control in older adults is mainly reflected in poor weight shifting abilities; in a recent study it was found that the primary cause of falling in elderly is incorrect weight shifting [4].

Standing is considered to be stable when the center of mass (COM) is within the boundaries of the base of support (BOS) [5]. The position of the COM is controlled by the position and changes in position of the center of pressure (COP) [5], [6]. To maintain balance during daily life activities, people need to shift their weight to control the position of the COM by adjusting their COP position in such a way that the COM stays within the BOS [5], [6]. In situations when balance is suddenly disturbed this control needs to be fast and accurate, to restore balance. However, for targeted movements, there is a trade-off between the movement duration and the accuracy demands of the movement. A goal-directed action can be performed quickly, but this will come at the expense of the accuracy and when the movement has high accuracy constraints, movement time will increase [7]. This trade-off is mainly studied in targeted movements of the upper extremities [8], but it is also found that the movement time for targeted movements of the whole body increased with increasing accuracy demands [9], [10].

Apparently it is a challenge to perform targeted COP movements quickly and accurately. Older adults may have even more difficulties with this than younger adults, due to the degeneration of sensory and musculoskeletal systems involved in balance control [3]. Previous research showed indeed an increased movement time and reduced control of the direction of body movement during rapid untargeted COP movements in elderly with increased age and fall-risk [11]. This age-related decrease in speed and accuracy was also found for targeted COP movements [6], [12]. By performing targeted movements with the whole body as fast and accurate as possible, in anterior and posterior direction to targets of different sizes and at different target distances, older women (76±6 years) moved slower than young women (23±3 years) and needed more corrective movements to maintain COP accuracy [12]. In addition, elderly undershot the target more often, which might be a sign of a decreased ability to perform accurate weight shifts to keep balance [6].

Elderly thus have more difficulties with performing fast and accuracy constrained whole body aiming movements, especially when the movement amplitude is larger and the target size smaller [6], [12]. This might be a result of difficulties with developing sufficient torques in the required muscles that come with age [13]. A limitation of previous discussed studies [6], [9], [10], [12] is that it was possible to plan the movement in advance, because the target position was predictable. In many daily life activities it is possible to anticipate on the upcoming movement. However, in situations when balance is suddenly disturbed anticipating on the next movement is more difficult, since the movements need to be fast to restore balance or to prevent from falling. In a previous study differences were found in the control of weight transfer between young and older adults when performing whole body aiming movements to either predictable or unpredictable targets. It was shown that young healthy adults performed faster and more fluent and accurate COP movements and that they benefitted more from the possibility to anticipatorily plan the movement than older adults [14].

So far, there is only one study [14] that examined COP movements to unpredictable targets. It is of great importance to further explore the understanding about weight shifting strategies in older adults when it is not possible to anticipatorily plan the upcoming movement. Therefore, differences in weight transfer strategies between young and older adults will be examined in this experiment. Young and older adults perform targeted COP movements by leaning in different directions to unpredictable targets at different distances and of different sizes. The aim of this study is to examine the movement time, fluency and accuracy of target directed whole body movements to unpredictable targets and to examine whether ageing effects have an influence on these three outcomes.

It is expected that both groups need more time to perform the movement and move less fluent and less accurate when the task is more difficult (i.e. smaller target size and longer target distance). In addition, it is hypothesized that the older adults need more time to perform the targeted COP movements and that their movements are less accurate and less fluent compared to younger adults, especially when the target size is smaller and the target distance is longer.

## Methods

### Ethics Statement

The local ethics committee of the Center of Human Movement Sciences of the University Medical Center Groningen approved the experiment.

### Participants

Nine healthy older adults (70.3±6.9 years) and twelve healthy young adults (20.9±0.5 years) participated in the study. The older adults were able to stand and walk without assistance and to perform targeted leaning movements with the whole body. All participants participated at least once a week in different sports activities, so they are considered relatively fit and active. Before the experiment started, all participants gave written informed consent.

### Equipment

**Figure 1:**
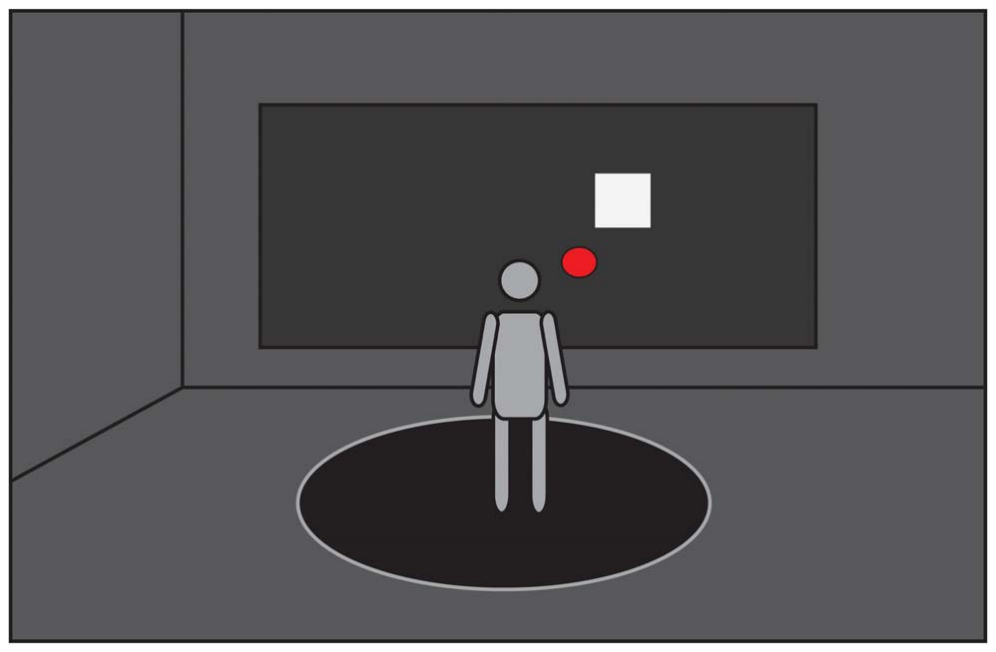
Experimental Setup. A person standing on the platform looking at the projection screen in front of him and performing the task by moving the red dot (COP position) to the white target, by shifting his weight as fast and accurate as possible.

**Figure 2:**
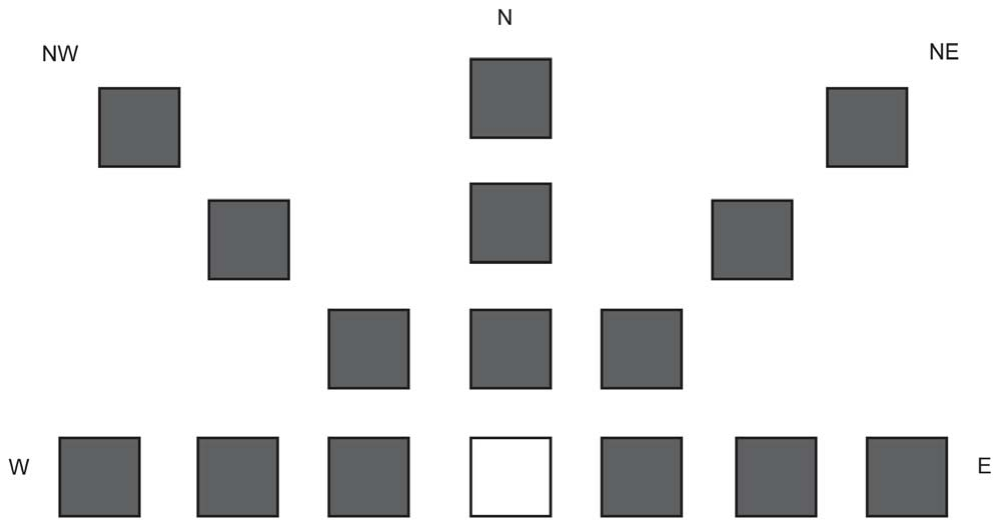
Schematic overview of all possible target positions. The white square is the home target, the grey squares are the goal targets. In reality only one target was visible during the task. The letters W, NW, N, NE and E indicate the different wind directions (west, northwest, north, northeast and east respectively).

The experiment was performed in the Computer Assisted Rehabiliation ENvironement (CAREN) lab in Groningen (Motek Medical BV, Amsterdam, the Netherlands). The CAREN system consists of a platform with a diameter of 2 meter with two integrated force plates (40×60 cm), where the participants stood on during the experiment. Forces and center of pressure (COP) position were collected with a sample frequency of 1000 Hz. On a large projection screen 2 meter in front of the subjects a simple game environment was projected, which was created using the D-flow software of Motek Medical BV. The game consisted of a red dot (Ø 0.115 m), which represented real time COP position after filtering the raw COP data with 10 Hz and a white block, which represented either the home target or one of the goal targets. Each trial a goal target, which varied in size, appeared at a random position. The participants had to move the red dot from the home target to the goal target and back to the home target as fast and accurate as possible by shifting their weight (Figure 1 and 2).

### Targets

To gain more insight in the control of weight shifting within the surface of support in older adults, the participants had to move the whole body in different directions, to move a cursor towards targets of different sizes and at different distances projected on a screen (Figure 2). Three target sizes, three target distances and five movement directions were determined.

The maximal distance from the home target to the goal targets was based on values of the limit of stability (LOS) in anterior direction found in previous research. The LOS is the maximum distance a person can displace his/her COM by leaning in a specific direction, without stepping or grasping to restore balance and without falling [15]. The maximal target distance in this study was based on the study of Prado et al. (2010) [16]. They found that the LOS of young adults (µ = 25 years) was 0.11 m in anterior-posterior direction and 0.31 m in medio-lateral direction. In addition they found that the LOS of older adults (µ = 70 years) is 30% percent lower in anterior-posterior direction and 10% lower in medio-lateral direction than the LOS of young adults [16]. Since the targets needed to be achievable for both young and older adults the LOS of young adults, with a correction of about 30% in anterior direction was taken as maximal distance for all directions. Consequently, the three target distances in terms of COP displacement were 0.08, 0.06 and 0.04 m corresponding with respectively 0.24, 0.36 and 0.48 m between the home and goal target on the screen. Target sizes were determined based on pilot measurements, which resulted in target sizes of 0.12×0.12 m, 0.18×0.18 m and 0.24×0.24 m on the screen. The size of the home target was always 0.18×0.18 m, the mean target size. The position of the home target was determined by the condition that the COP felt in the home target during quiet stance.

During each trial the goal target appeared in one of the 5 different wind directions (W, NW, N, NE, E). The participants had to lean in sideward, diagonally forward en forward direction. Leaning in backward direction was not included, since it is perceptually difficult and due to an increased fall risk when leaning backwards, participants then had to wear a safety harness, which might have hampered the leaning movements in all directions. The three sizes, three target distances en five target directions resulted in 45 unique trials that were performed by the participants (Figure 2).

### Procedure

Before the experiment started, participants practiced with moving the red dot in all directions, to get familiarized with the experimental setup. In order to clarify the task for elderly, they were told that moving the body in forward direction resulted in an upward movement of the red dot and that moving the body in backward direction resulted in a downward movement of the red dot on the screen.

After the familiarization, participants were asked to stand with each foot on a force plate, two meters in front of the screen. Subsequently, participants were asked to keep the red dot as still as possible for twenty seconds to calibrate COP position, this calibration was repeated before every trial. After the calibration, the experiment started. Each trial, subjects began with the red dot on the home target. When the red dot was on the home target for 0.5 second, a goal target appeared and the participants were instructed to move the red dot from the home target to the goal target as fast and accurate as possible. The goal target disappeared and the home target reappeared if the red dot was on the goal target for 0.5 second. As soon as the red dot was repositioned on the home target for 0.5 second, a new goal target appeared. The goal targets appeared randomly, so participants were not able to anticipatorily plan their goal-directed movement in advance.

The experimental trials were performed in five blocks of 45 trials; consequently each participant reached each unique target five times. Between the blocks there was enough time to rest, to avoid that fatigue would affect the results.

### Data Analysis

**Figure 3:**
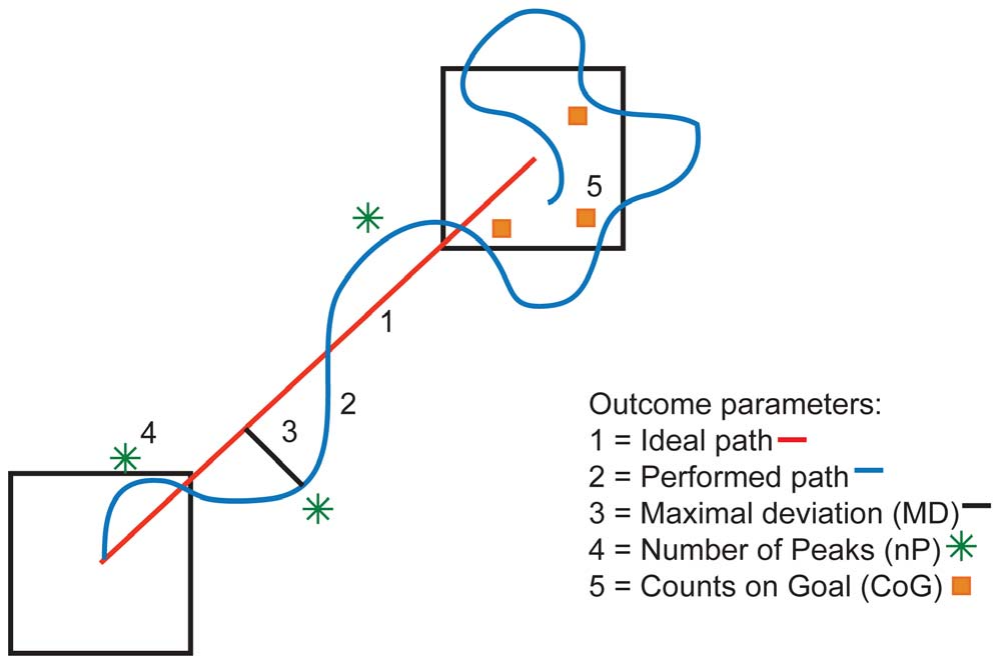
Schematic overview of the outcome parameters.

Raw COP data was filtered with a 5 Hz low-pass 4th order Butterworth filter. Five outcome measures were calculated using the force plates data. Movement time (MT) was calculated as the time between the appearance of the goal target and the moment the participant reached the goal target, where reaching the goal target means that the COP stayed in the goal target for 0.5 second. Fluency of the movement was expressed by two measures; the maximal deviation (MD) of the performed path with respect to the ideal path and the number of peaks (nP), or inflections in the performed path (Figure 3). Movement accuracy was expressed by calculating two measures; the number of times the cursor hit the goal target before a target switch was realized (i.e. the cursor had to stay for 0.5 second in the goal target) as called Counts on Goal (CoG) and the time required to realize a target switch after the goal target was hit by the cursor for the first, as called dwelling time (DT).

A repeated measures ANOVA was performed in IBM SPSS Statistics 20 with the main factor size and distance as within factors and age group as between factor. Interaction effect of distance*age, size*age, distance*size and distance*size*age were assessed. When the assumption of Mauchly's sphericity was not met, the Huynh-Feldt correction was used to correct for the degrees of freedom.

## Results

**Figure 4:**
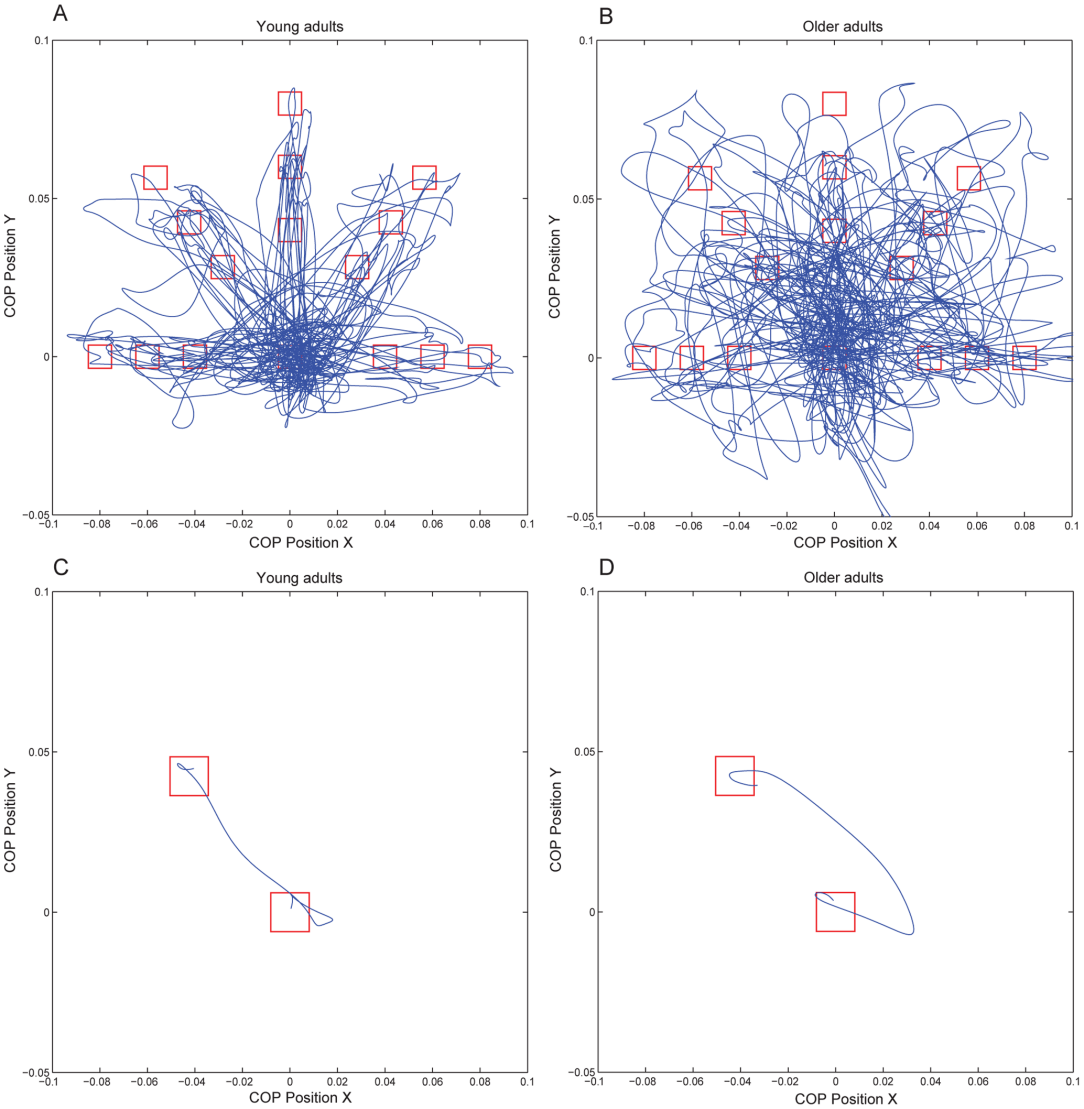
COP trajectories of one block of 45 experimental trials of a young subject (A) and an older subject (B) and COP trajectory of one experimental trial of a young subject (C) and an older subject (D); COP position in x direction is plotted against COP position in y direction.

Figure 4 shows the COP trajectories of a representative young and older subject for one block of 45 leaning movements (Figure 4A and 4B) to the targets in different directions, of different sizes and at different distances and for one experimental trial (Figure 4C and 4D). Table 1 shows the means and standard deviations of all outcome measures for all conditions. Statistics are presented in table 2.

**Table 1:** Mean and Standard Deviation of Movement Time (MT), number of peaks in the performed path (nP), maximal deviation of the performed path with respect to the ideal path (MD), the amount of times the cursor hit the goal target (CoG) and the dwelling time around the goal target (DT), for young (Y) and elderly (E) subjects.

		Distance 1			Distance 2			Distance 3		
	Age	Size 1	Size 2	Size 3	Size 1	Size 2	Size 3	Size 1	Size 2	Size 3
**MT (s)**	Y	1.59 (.21)	1.43 (.15)	1.27 (.07)	1.74 (.20)	1.68 (.51)	1.43 (.07)	2.02 (.24)	1.84 (.22)	1.61 (.09)
	E	1.72 (.11)	1.49 (.12)	1.31 (.12)	2.07 (.27)	1.78 (.16)	1.54 (.13)	2.86 (.33)	2.30 (.12)	1.94 (.19)
**nP**	Y	4.26 (.68)	3.87 (.48)	3.41 (.37)	4.60 (.61)	4.04 (.59)	3.65 (.31)	5.57 (.95)	4.91 (.55)	4.32 (.46)
	E	5.28 (.40)	4.49 (.31)	3.81 (.41)	6.26 (.94)	5.26 (.41)	4.51 (.32)	8.47 (1.01)	6.94 (.50)	5.89 (.63)
**MD (cm)**	Y	1.81 (.32)	1.72 (.28)	1.68 (.31)	1.79 (.29)	1.72 (.33)	1.77 (.28)	1.99 (.37)	2.00 (.28)	1.83 (.29)
	E	2.06 (.27)	2.05 (.28)	1.93 (.28)	2.44 (.56)	2.23 (.30)	2.10 (.34)	2.68 (.54)	2.63 (.46)	2.58 (.47)
**CoG**	Y	1.49 (.35)	1.32 (.25)	1.17 (.13)	1.44 (.24)	1.21 (.14)	1.14 (.13)	1.49 (.29)	1.38 (.22)	1.19 (.13)
	E	1.65 (.19)	1.41 (.26)	1.26 (.14)	1.68 (.33)	1.42 (.22)	1.27 (.22)	2.01 (.26)	1.69 (.29)	1.38 (.14)
**DT (s)**	Y	.82 (.24)	.68 (.18)	.54 (.09)	.82 (.14)	.67 (.09)	.62 (.08)	.92 (.20)	.80 (.14)	.69 (.09)
	E	.91 (.11)	.72 (.17)	.58 (.12)	1.00 (.22)	.81 (.14)	.66 (.12)	1.39 (.29)	1.05 (.18)	.82 (.12)

**Table 2:** F-values of main effects (Age, Distance and Size) and interaction effects (Distance*Age and Size*Age), for Movement Time (MT), number of peaks in the performed path (nP), maximal deviation of the performed path with respect to the ideal path (MD), the amount of times the cursor hit the goal target (CoG) and the dwelling time around the goal target (DT).

	Age	Size	Distance	Size*Age	Distance*Age
**MT**	F(1,19) = 21.22***	F(2,38) = 90.87***	F(2,38) = 152.71***	F(2,38) = 8.06**	F(2,38) = 21.91***
**nP**	F(1,19) = 55.57***	F(2,38) = 113.48***	F(2,38) = 276.51***	F(2,38) = 12.64***	F(2,38) = 47.25***
**MD**	F(1,19) = 13.66**	F(2,38) = 12.86***	F(2,38) = 52.21***	F(2,38) = 1.15 NS	F(2,38) = 13.10***
**CoG**	F(1,19) = 9.08**	F(2,38) = 68.33***	F(2,38) = 16.17***	F(2,38) = 3.53*	F(2,38) = 6.53**
**DT**	F(1,19) = 10.48**	F(2,38) = 94.90***	F(2,38) = 44.65***	F(2,38) = 6.53**	F(2,38) = 9.88***

* = p<.05.

** = p<.01.

*** = p<.001, NS =  not significant.

### Target Size and Distance

**Figure 5:**
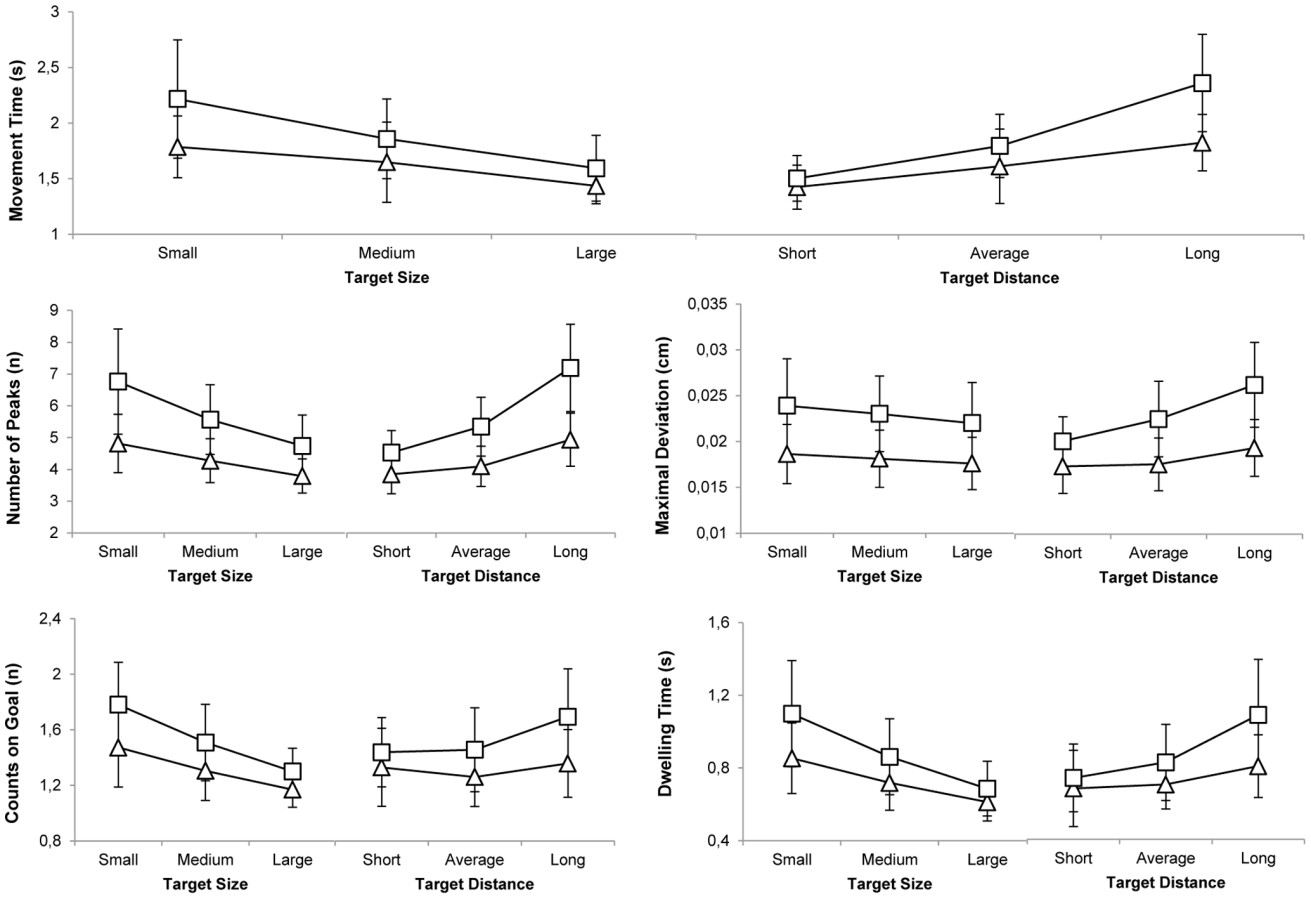
Mean values for all outcome measures for the young and older adults group (triangles, squares respectively) for the three different target sizes and target distances. Error bars represent group standard deviations. Averaged over sub values and standard deviations of all outcome measures for each size and distance.

There were significant main effects of target size and target distance on all outcome measures (table 2). Movement Time (MT) significantly increased with decreasing target size and with increasing target distance. The fluency of the movement significantly decreased with decreasing target size and increasing target distance, which was expressed in an increased number of peaks in the performed path and an increased maximal deviation of the performed path. The accuracy of the movement significantly decreased with decreasing target size and increasing target distance, which was expressed in more hits on the goal target and a longer dwelling time (Figure 5). These results imply that older adults needed more time to perform the movement and moved less fluent and less accurate compared to younger adults, when the leaning task became more difficult (i.e. smaller target size and longer target distance).

### Age

There was a significant main effect of age on all outcome measures (table 2). Movement time significantly increased with increasing age. Older adults moved less fluent, which was expressed in more peaks in the performed path and a larger maximal deviation of the performed path. In addition, elderly moved less accurate, which was expressed in more hits on the goal target and a longer dwelling time (Figure 5). These results imply that older adults needed more time to perform the whole-body aiming movements and moved less accurate and fluent compared to younger adults.

### Size*Age and Distance*Age

There were significant interaction effects of distance*age and size*age for all outcome measures, except for the maximal deviation, where no significant interaction effect was found for size*age (table 2). The effect of distance and size on the speed, fluency and accuracy of the movement significantly increased with increasing age (Figure 5).

## Discussion

The aim of this was to gain more insight in the differences in postural control strategies between young and older adults during a postural control task requiring aiming movements of the whole body towards unpredictable targets. Young and older adults performed a weight-shifting task by moving the whole body in different directions to move a cursor towards targets of different sizes and at different distances projected on a screen in front of them. The task required that the subjects controlled their weight shifting quickly and accurately to reach a target location. The target appeared at an unpredictable location, so people had little time to anticipatory plan the aiming movement to the goal target in advance. It was expected that the participants needed more time to perform the COP movements and moved less fluently and less accurately when the task was more difficult (i.e. smaller target size and longer target distance) and that this would be more pronounced in older adults compared to younger adults.

The results clearly showed that, with increasing task difficulty, both groups had more difficulties with executing the targeted whole body movements quickly, fluently and accurately. When the target distance increased and the target size decreased, the movement time increased, the movement trajectory was more irregular and more time was spend dwelling around the goal target, which is in line with previous studies[9], [10]. These results indicate that people use a more variable and slower postural control strategy when they have to execute whole-body aiming movements with larger amplitudes and with higher accuracy constraints. This is consistent with the trade-off between speed and accuracy of a movement found in targeted reaching movements of the upper extremity [8]. This trade-off predicts that with increasing accuracy demands, movement time increases [7]. The present study showed that this relationship is not unique for upper extremity reaching movements, but does also hold for tasks that require whole body aiming movements when standing. During many daily life activities it is required to quickly and accurately control COP position, to control the COM position with respect to the boundaries of the BOS and thus to remain stable. When the COP needs to be shifted quickly, for example to adapt to a sudden perturbation in daily life, this happens at the expense of the accuracy of the COP movement. Consequently, it is possible that the COM exceeds the boundaries of the BOS and stability is no longer maintained.

Particularly for elderly, this might have implications for postural control and fall risk in daily life. This agrees with the results that in addition to the task effects a significant effect of age was observed, i.e. older adults had more difficulties with performing the whole body aiming movements rapidly, fluently and accurately, compared to younger adults. More specifically, older adults needed more time to perform the aiming task, showed larger deviations from the ideal path and more inflections and peaks in the performed path than younger adults. In addition, compared to younger adults, older adults hit the target more often and dwelled around the goal target for a longer time before they actually landed and stayed in the goal target for 0.5 second. These results imply that older adults had more difficulties with stabilizing their posture at a given position compared to younger adults. Moreover, older adults needed even more time and were less accurate and fluent in the performance of the weight shifting task compared to younger adults when the target size was smaller and the target distance longer, as was indicated by significant interaction effects between size*age and distance*age. The findings of the present study are in line with results of previous studies [6], [8], [12] that showed that elderly moved slower and with more sub movements compared to younger adults in a target directed reaching task with the upper extremity [8] and the whole body [6], [12], especially with increasing task difficulty [8]. These results indicate that older adults have more difficulties with executing a stable and quick postural control strategy compared to younger adults. The increased movement time, longer dwelling time and overshoot distance might be indicative for an increased fall risk in elderly. In quiet stance, a greater amount of postural sway is associated with a history of falls in older adults [17] and increased postural reaction time is associated with increased fall risk in elderly [18]. In addition, the results indicate that elderly might have more difficulties with executing an adequate (quick and accurate) adaptation to a perturbation in daily life.

Older adults thus exhibit slower and more variable postural control strategies compared to younger adults in a weight shifting task, when they have less time to anticipatorily plan the upcoming movement. A possible explanation for these differences between elderly and younger adults may be found in cognitive declines that come with age. Previous research in stepping abilities showed that elderly need more time than younger adults to look at a target and plan and execute an accurate step [19], [20]. These results indicate age-related delays in the processing of visuomotor information to guide a goal-directed movement [19], [20]. These delays in visuomotor processing times may in the present study have influenced the timing and execution of the weight shifting task in elderly, leading to an increased movement time and less smooth COP movements. In the present study the participants were forced to predominantly use ‘online’ visuomotor information to guide the reaching movement, since the target position was unpredictable. Therefore, the increased movement time found in older adults could also be explained by age-related differences in the ability to use this visuomotor information while executing movements. Previous research showed that elderly, when forced to use only online visuomotor information, exhibit greater delays in their movements and more motor errors compared to younger adults [21]. The delay in the use of online visuomotor information may have resulted in a slower and more variable task performance. Elderly probably tried to compensate for deviations from the intended trajectory while aiming to the goal target. Due to the delay in the use of online visuomotor information in elderly, each compensation was executed with a delay, what could have resulted in more variable COP trajectories.

Differences in the ability to produce appropriate anticipatory postural adjustments (APAs), due to age-related declines in proprioception and sensory and motor performance [3], [22], might also explain the differences found in weight shifting strategies between young and older adults. A number of studies have shown that APAs play a role in the actual execution of goal-directed movements [23], [24] and that the mechanisms for producing APAs alter with age [2], [25]–[27]. APAs are produced prior to movement onset and are reflected by recruitment of postural muscles and by displacements of the COP [24], [25]. APAs are found to be delayed in elderly while performing fast voluntary arm raising movements [25]. In the present study, the participants were instructed to perform the reaching movement as fast and accurate as possible after the target became visible. When the production of an APA is delayed, time to movement onset will increase, which might in the present study have contributed to the increased movement time found in older adults. In addition, the participants had little time to anticipatorily plan the upcoming movement, since the target position was unpredictable. Therefore, it might be possible that the older adults were not able to produce adequate APAs before movement onset and therefore had to correct by making compensatory adjustments during the movement itself [27]. The inability to produce adequate APAs and consequently the need for compensatory adjustments during the weight shifting movement might be reflected in the increased variability in the COP trajectories of the older adults found in the present study.

The results of this study showed that the ability to quickly and accurately control weight shifting is reduced in elderly. In this view, training the temporal and spatial aspects of postural control might be useful to decrease fall risk in elderly. A previous study showed that elderly women improved balance function after receiving a balance training where the participants learned to control the movement of the COP during dynamic weight shifting, with varying spatial and temporal demands [28]. Videogames that incorporate training, so called ‘exergames’, may be even more promising in enhancing postural control in elderly, since they provide continuous visual feedback and the combination of gaming and exercise is enjoyable. Previous studies indeed showed that postural control of elderly improved after an exergaming training intervention [29], [30]. Considering the results of the present study, an exergame that require quick and accurate weight shifts might thus be useful to increase postural control in elderly and consequently decrease fall risk.

It is important to realize that all participants in this study were relatively fit and healthy. The older adults lived independently and walked without support. Furthermore, both young and elderly could fulfill the leaning task well within their limits of stability, indicating that this weight-shifting task was not a serious challenge. Yet, despite this, this study revealed distinct differences in weight shifting strategies between young and older adults. To gain more insight in the consequences of these age-related differences in weight shifting it would be interesting to examine differences in postural control strategies in more critical situations, such as a near fall. In addition it would be interesting to include even older people, in order to examine how and when postural control strategies change with age.

The results of this study provided insight in the age-related differences in the control of weight shifting when the movement cannot be anticipatorily planned in advance. It was shown that older adults were slower, less fluent and less accurate compared to younger adults, in performing a weight shifting task, especially with increasing task difficulty. This weight-shifting strategy seems characterizing for an increased fall risk in elderly, since the results indicate that elderly might have more difficulties with executing an adequate (quick and accurate) adaptation to a perturbation in daily life. Future research should include older participants and should examine postural control in elderly in more challenging settings. This knowledge can be used in developing interventions to train balance in elderly, which should eventually result in lower fall rates in elderly.
